# Efficient Selection of New Immunobiotic Strains With Antiviral Effects in Local and Distal Mucosal Sites by Using Porcine Intestinal Epitheliocytes

**DOI:** 10.3389/fimmu.2020.00543

**Published:** 2020-04-08

**Authors:** Leonardo Albarracin, Valeria Garcia-Castillo, Yuki Masumizu, Yuhki Indo, Md Aminul Islam, Yoshihito Suda, Apolinaria Garcia-Cancino, Hisashi Aso, Hideki Takahashi, Haruki Kitazawa, Julio Villena

**Affiliations:** ^1^Laboratory of Immunobiotechnology, Reference Centre for Lactobacilli (CERELA-CONICET), Tucuman, Argentina; ^2^Food and Feed Immunology Group, Laboratory of Animal Products Chemistry, Graduate School of Agricultural Science, Tohoku University, Sendai, Japan; ^3^Laboratory of Bacterial Pathogenicity, Faculty of Biological Sciences, University of Concepción, Concepción, Chile; ^4^Department of Food, Agriculture and Environment, Miyagi University, Sendai, Japan; ^5^Cell Biology Laboratory, Graduate School of Agricultural Science, Tohoku University, Sendai, Japan; ^6^Livestock Immunology Unit, International Education and Research Center for Food Agricultural Immunology (CFAI), Graduate School of Agricultural Science, Tohoku University, Sendai, Japan; ^7^Laboratory of Plant Pathology, Graduate School of Agricultural Science, Tohoku University, Sendai, Japan; ^8^Plant Immunology Unit, International Education and Research Centre for Food and Agricultural Immunology (CFAI), Graduate School of Agricultural Science, Tohoku University, Sendai, Japan

**Keywords:** porcine intestinal epithelial cells, TLR3, *Lactobacillus rhamnosus* CRL1505, *Lactobacillus plantarum* MPL16, antiviral response, respiratory immunity

## Abstract

Previously, we evaluated the effect of the immunobiotic strain *Lactobacillus rhamnosus* CRL1505 on the transcriptomic response of porcine intestinal epithelial (PIE) cells triggered by the challenge with the Toll-like receptor 3 (TLR-3) agonist poly(I:C) and successfully identified a group of genes that can be used as prospective biomarkers for the screening of new antiviral immunobiotics. In this work, several strains of lactobacilli were evaluated according to their ability to modulate the expression of *IFN*α, *IFN*β, *RIG1*, *TLR3*, *OAS1*, *RNASEL*, *MX2*, *A20*, *CXCL5, CCL4*, *IL-15*, *SELL*, *SELE*, *EPCAM*, *PTGS2*, *PTEGES*, and *PTGER4* in PIE cells after the stimulation with poly(I:C). Comparative analysis of transcripts variations revealed that one of the studied bacteria, *Lactobacillus plantarum* MPL16, clustered together with the CRL1505 strain, indicating a similar immunomodulatory potential. Two sets of *in vivo* experiments in Balb/c mice were performed to evaluate *L. plantarum* MPL16 immunomodulatory activities. Orally administered MPL16 prior intraperitoneal injection of poly(I:C) significantly reduced the levels of the proinflammatory mediators tumor necrosis factor α (TNF-α), interleukin 6 (IL-6), and IL-15 in the intestinal mucosa. In addition, orally administered *L. plantarum* MPL16 prior nasal stimulation with poly(I:C) or respiratory syncytial virus infection significantly decreased the levels of the biochemical markers of lung tissue damage. In addition, reduced levels of the proinflammatory mediators TNF-α, IL-6, and IL-8 were found in MPL16-treated mice. Improved levels of IFN-β and IFN-γ in the respiratory mucosa were observed in mice treated with *L. plantarum* MPL16 when compared to control mice. The immunological changes induced by *L. plantarum* MPL16 were not different from those previously reported for the CRL1505 strain in *in vitro* and *in vivo* studies. The results of this work confirm that new immunobiotic strains with the ability of stimulating both local and distal antiviral immune responses can be efficiently selected by evaluating the expression of biomarkers in PIE cells.

## Introduction

More than a third of child deaths worldwide are attributed to malnutrition and its profound impact on the immune system and the host resistance to infections. In this sense, pneumonia and diarrhea remain the leading causes of death of children. Together, these two infectious diseases account for 29% of all deaths of children younger than 5 years and result in the loss of 2 million young lives annually (1). In 2009, the World Health Organization (WHO) separately published two strategies for the control of pneumonia and diarrhea. Since these strategies were implemented globally, it has been recognized that pneumonia and diarrhea could be treated more effectively in a coordinated manner. Thus, WHO-UNICEF published in 2013 an Integrated Global Action Plan for the Prevention and Control of Pneumonia and Diarrhea (GAPPD), which proposes a cohesive approach to ending deaths from preventable pneumonia and diarrhea in children (1). The GAPPD proposes interventions to create healthy environments, promotes practices to protect children from infectious diseases, and ensures that all children have access to appropriate preventive and treatment measures. The document emphasizes the importance of healthy nutrition to reduce the incidence and severity of pneumonia and diarrhea simultaneously.

Various clinical trials and studies in animal models have demonstrated the ability of probiotic lactic acid bacteria (LAB) with immunomodulatory activities, also known as immunobiotics, to improve the resistance to intestinal viral infections (2, 3). Although most of the research related to the beneficial effect of immunobiotics on the host’s immune system has focused on the stimulation of intestinal immunity to protect against viral infections (4), it has also been shown that the oral administration of some immunobiotics strains can beneficially modulate not only the local intestinal immunity but also immune responses in distant mucosal sites such as the respiratory tract (5, 6). Then, it is believed that appropriate immunobiotic LAB strains can be effectively used as preventive therapies to improve antiviral defenses and reduce the complications from the deregulated inflammatory responses in both the intestine and the respiratory tract. Therefore, the efficient selection of immunobiotic strains that orally administered are capable of favorably modulating intestinal and respiratory antiviral immunity could contribute to reducing the risk of viral infections, having a simultaneous impact on resistance to pneumonia and diarrhea.

*Lactobacillus rhamnosus* CRL1505, orally administered to mice, is able to differentially modulate both intestinal (7, 8) and respiratory (9, 10) antiviral immunity. Moreover, in a randomized controlled trial conducted in children younger than 5 years, it was demonstrated that the immunobiotic CRL1505 strain is capable to enhance mucosal immunity and reduced the incidence and severity of intestinal and respiratory infections (2). Of note, our studies in mice models have clearly shown that the immunomodulatory capacities of *L. rhamnosus* CRL1505 are a strain-specific property because other immunobiotic strains such as *Lactobacillus plantarum* CRL1506 can stimulate only the intestinal immunity after their oral administration (7–10).

It is well known that intestinal epithelial cells (IECs) had a central role in determining the type of immune response triggered by antigens in the intestinal mucosa through their interactions with immune cells (11, 12). In this regard, studies have reported that immunomodulatory LAB modulate intestinal immune responses by regulating the functions of IECs (13). Therefore, we hypothesized that transcriptomic analysis evaluating the effect of *L. rhamnosus* CRL1505 and *L. plantarum* CRL1506 on IECs could bring valuable information about their capacity of modulating the intestinal innate antiviral immune response, as well as provide some clues about their differential ability to influence immunity in the respiratory tract. By using porcine intestinal epithelial (PIE) cells that express the pattern recognition receptor (PRR) Toll-like receptor 3 (TLR-3) and respond to poly(I:C) stimulation (14, 15); we studied the similarities and differences of *L. rhamnosus* CRL1505 and *L. plantarum* CRL1506 in terms of their ability to modulate the immunotranscriptomic response of epithelial cells (16). The transcriptional profiling performed in PIE cells allowed us to characterize the immune and immune-related genes involved in their response to TLR3 activation, which included type I interferons (IFNs), antiviral factors, cytokines, chemokines, adhesion molecules, enzymes involved in prostaglandin biosynthesis, PRRs, and negative regulators of the TLR signaling pathway. In addition, we were able to confirm that the CRL1505 and CRL1506 strains differently regulated the immunotranscriptomic response of poly(I:C)-challenged PIE cells (16). Quantitative and qualitative differences in the expression of immune and immune-related genes in PIE cells were found when *L. rhamnosus* CRL1505 and *L. plantarum* CRL1506 treatments were compared. Moreover, the comparative analysis of the effect of the two strains allowed us to identify a group of genes that could be used as potential biomarkers for the efficient selection of new antiviral immunobiotics in PIE cells, which could beneficially influence both the intestinal and the respiratory antiviral immunity.

In this work, we tested this hypothesis by evaluating the influence of several immunomodulatory and non-immunomodulatory lactobacilli strains in the expression of *IFN*α, *IFN*β, *RIG1*, *TLR3*, *OAS1*, *RNASEL*, *MX2*, *A20*, *CXCL5, CCL4*, *IL-15*, *SELL*, *SELE*, *EPCAM*, *PTGS2*, *PTEGES*, and *PTGER4* in poly(I:C)-challenged PIE cells and by studying *in vivo* the effect of orally administered lactobacilli on the intestinal and respiratory antiviral innate immune responses.

## Materials and Methods

### PIE Cells

The PIE cell line was originally derived from intestinal epithelia isolated from an unsuckled neonatal swine (17). Porcine intestinal epithelial cells are intestinal non-transformed cultured cells that assume a monolayer with a cobblestone and epithelial-like morphology and with close contact between cells during culture (14, 17, 18). Porcine intestinal epithelial cells were maintained in Dulbecco modified eagle medium (DMEM) (Invitrogen Corporation, Carlsbad, CA, United States) supplemented with 10% fetal calf serum, 100 mg/mL streptomycin and 100 U/mL penicillin at 37°C in an atmosphere of 5% CO_2_ (16, 18–20).

### Microorganisms

*Lactobacillus rhamnosus* CRL1505, *L. plantarum* CRL1506, *L. rhamnosus* CRL576, and *L. plantarum* CRL681 belong to CERELA Culture Collection; *L. rhamnosus* IBL07 belongs to the Infection Biology Laboratory of IMMCA-CONICET-UNT and was kindly given by Dr. Vizoso-Pinto. *Lactobacillus plantarum* MPL16 belongs to the Food and Feed Immunology Group Culture Collection, These strains were grown in Man–Rogosa–Sharpe broth at 37°C. For the *in vitro* immunomodulatory assays, overnight cultures were harvested by centrifugation, washed three times with sterile phosphate-buffered saline (PBS), counted in a Petroff–Hausser counting chamber, and resuspended in DMEM until use.

### Immunomodulatory Effect of Lactobacilli in PIE Cells

The study of the immunomodulatory capacity of lactobacilli was performed in PIE cells as described previously (15, 16, 18, 20). Porcine intestinal epithelial cells were seeded at 3 × 10^4^ cells per well in 12-well type I collagen-coated plates (Sumitomo Bakelite Co., Tokyo, Japan) and cultured for 3 days. After changing medium, lactobacilli (5 × 10^8^ cells/mL) were added, and 48 h later, each well was washed vigorously with medium at least three times to eliminate all stimulants. Then cells were stimulated with poly(I:C) (60 μg/mL) for 12 h for reverse transcription–polymerase chain reaction (RT-PCR) studies.

### Quantitative Expression Analysis by Two-Step Real-Time Quantitative PCR

Two-step real-time quantitative PCR (qPCR) was performed to characterize the expression of selected genes in PIE cells as described previously (16). TRIzol reagent (Invitrogen) was used for total RNA isolation from each PIE cell sample, and Quantitect RT kit (Qiagen, Tokyo, Japan) was used for the synthesis of all cDNAs according to the manufacturer’s recommendations. Real-time qPCR was carried out using a 7300 real-time PCR system (Applied Biosystems, Warrington, United Kingdom) and the Platinum SYBR green qPCR SuperMix uracil-DNA glycosylase with 6-carboxyl-X-rhodamine (ROX) (Invitrogen). The primers used for the study of *IFN*α, *IFN*β, *RIG1*, *TLR3*, *OAS1*, *RNASEL*, *MX2*, *A20*, *CXCL5, CCL4*, *IL-15*, *SELL*, *SELE*, *EPCAM*, *PTGS2*, *PLA2G4A*, *PTEGES*, and *PTGER4* expressions in this study were described before (16, 20, 21). The PCR cycling conditions were 2 min at 50°C, followed by 2 min at 95°C, and then 40 cycles of 15 s at 95°C, 30 s at 60°C, and 30 s at 72°C. The reaction mixtures contained 5 μL of sample cDNA and 15 μL of master mix, which included the sense and antisense primers. According to the minimum information for publication of quantitative real-time PCR experiments guidelines, β-actin was used as a housekeeping gene because of its high stability across porcine various tissues (14, 15, 21). Expression of β-actin was used to normalize cDNA levels for differences in total cDNA levels in the samples.

### Animals, Feeding Procedures, and Poly(I:C) Challenge

Male 6-week-old BALB/c mice were obtained from the closed colony kept at CERELA-CONICET. Animals were housed in plastic cages in a controlled atmosphere (22°C ± 2°C temperature, 55% ±2% humidity) with a 12-h light/dark cycle. Lactobacilli were orally administered to different groups of mice for five consecutive days at a dose of 10^8^ cells/mouse per day in the drinking water (22, 23). The treated groups and the untreated control mice were fed a conventional balanced diet *ad libitum*.

Two sets of experiments were performed in treated and control mice. In the first set of experiments, mice were challenged by the intraperitoneal route with 100 μL of PBS containing 30 μg poly(I:C) according to our previous publication (8). Biochemical markers of injury as well as intestinal cytokines’ concentrations were evaluated 2 days after poly(I:C) administration as described below. In the second set of experiments, mice were lightly anesthetized, and 100 μL of PBS, containing 250 μg poly(I:C) (equivalent to 10 mg/kg body weight), was administered via the nares according to our previous publication (7). Mice received three doses of poly(I:C) with 24-h rest period between each administration.

### Respiratory Syncytial Virus Infection

Infection with human respiratory syncytial virus (RSV) strain A2 was performed as described previously (9). Briefly, RSV was grown in Vero cells for 3 h at 37°C, 5% CO_2_ at multiplicity of infection of 1 in 5 mL of DMEM. After cell lysis, virus supernatant was sucrose density gradient purified and stored in 30% sucrose at −80°C. For infection, mice were lightly anesthetized with isoflurane and intranasally challenged with 3.1 × 10^6^ plaque forming units (PFU) of RSV (9).

For the evaluation of viral infection, the RSV immunoplaque assay was performed (9). In brief, lung tissue was removed from mice, homogenized using a pellet pestle, and centrifuged at 2,600 × *g* for 10 min at 4°C to clarify supernatant. Serial dilutions of lung tissue–clarified supernatants were added into fresh Vero cells monolayers and incubated at 37°C, 5% CO_2_ for 3 h. All samples were run in triplicate. After incubation and removal of supernatant, 1 mL of fresh DMEM medium containing 10% fetal bovine serum, 0.1% penicillin-streptomycin, and 0.001% ciprofloxacin was added to monolayers. When extensive syncytia developed, monolayers were fixed with 1 mL of ice-cold acetone:methanol (60:40). Then, wells were treated with primary RSV anti-F (clones 131-2A; Chemicon, Temecula, CA, United States) and anti-G{mouse monoclonal [8C5 (9B6)] to RSV glycoprotein; Abcam} antibodies for 2 h, followed by secondary horseradish peroxidase anti–mouse immunoglobulin antibody (anti–mouse immunoglobulin G, horseradish peroxidase–linked antibody #7076; Cell Signaling Technology, Danvers, MA, United States) for 1 h. Plates washed twice with PBS containing 0.5% Tween 20 (Sigma, St. Louis, MO, United States) after each antibody incubation step. Individual plaques were developed using a DAB substrate kit (ab64238; Abcam, Cambridge, United Kingdom) following the manufacturer’s specifications. Results were expressed as log10 PFU/g of lung.

### Ethics Statement

Animals were housed in plastic cages and environmental conditions were kept constant, in agreement with the standards for animal housing. Animal welfare was in charge of researchers and special staff trained in animal care and handling at CERELA. The minimal number of animals required for an appropriate statistical analysis was calculated with the help of the Biostatistics Laboratory of CERELA.

Animals were housed individually during the experiments. All efforts were made to minimize the number of animals and their suffering. Animal health and behavior were monitored twice a day. Animals were euthanized immediately after the time point was reached by using xylazine and ketamine. No signs of discomfort or pain and no deaths were observed before mice reached the endpoints.

All experiments were carried out in compliance with the Guide for Care and Use of Laboratory Animals and approved by the Ethical Committee of Animal Care at CERELA, Argentina (protocol no. BIOT-CRL/14 and BIOT-CRL/11) (7, 8).

### Markers of Injury

Lactate dehydrogenase (LDH) and aspartate aminotransferase (AST) activities were determined in the serum to evaluate general toxicity of poly(I:C) in mice challenged by the intraperitoneal injection. Blood samples were obtained through cardiac puncture under anesthesia. LDH and AST activities, expressed as units per liter of serum, were determined by measuring the formation of the reduced form of nicotinamide adenine dinucleotide using the Wiener reagents and procedures (Wiener Lab, Buenos Aires, Argentina) (8).

Albumin content, a measure to quantitate increased permeability of the bronchoalveolar–capillarity barrier, and LDH activity, an indicator of general cytotoxicity, were determined in bronchoalveolar lavage (BAL) fluid. Bronchoalveolar lavage samples were obtained as described previously (7, 15). Briefly, the trachea was exposed and intubated with a catheter, and two sequential lavages were performed by injecting sterile PBS in each mouse lung. The recovered fluid was centrifuged for 10 min at 900 × *g*; the pellet was discarded, and the fluid was frozen at −70°C for subsequent analyses. Albumin content was determined colorimetrically based on albumin binding to bromocresol green using an albumin diagnostic kit (Wiener Lab). Lactate dehydrogenase activity, expressed as units per liter of BAL fluid, was determined by using the Wiener reagents and procedures (Wiener Lab) (7).

Lung wet-to-dry weight ratio was measured as previously described (7, 9). Wet-to-dry weight ratio was calculated as an index of intrapulmonary fluid accumulation, without correction for blood content.

### Cytokine Concentrations

Serum and BAL samples were obtained as described before (9). Briefly, blood samples were obtained by cardiac puncture under anesthesia. For BAL samples, the trachea was exposed surgically and intubated with a catheter. A small incision was made in the trachea, and two sequential lavages were performed in each mouse by injecting sterile PBS with 1% heparin. The recovered fluid was centrifuged for 10 min at 300 revolutions/min, and the supernatant was recovered. Intestinal fluid samples were obtained according to our previous publication (8). Briefly, the small intestine was flushed with 5 mL of PBS, and the fluid was centrifuged (10,000 × *g*, 4°C 10 min) to separate particulate material. The serum, BAL and intestinal supernatant samples were kept frozen at −80°C until use.

Tumor necrosis factor α (TNF-α), interleukin 6 (IL-6), IL-10, IL-15, IFN-β, and IFN-γ concentrations in serum, intestinal fluid, and BAL samples were measured with commercially available enzyme-linked immunosorbent assay technique kits following the manufacturer’s recommendations (R&D Systems, Minneapolis, MN, United States).

### Statistical Analysis

Statistical analyses were performed using GLM and REG procedures available in the SAS computer program (SAS, 1994). Comparisons between mean values were carried out using one-way analysis of variance and Fisher least significant difference test. For these analyses, *P* < 0.05 and *P* < 0.01 were considered significant.

## Results

### Modulation of TLR3-Induced Immunotranscriptome Changes in PIE Cells by Lactobacilli

**FIGURE 1:**
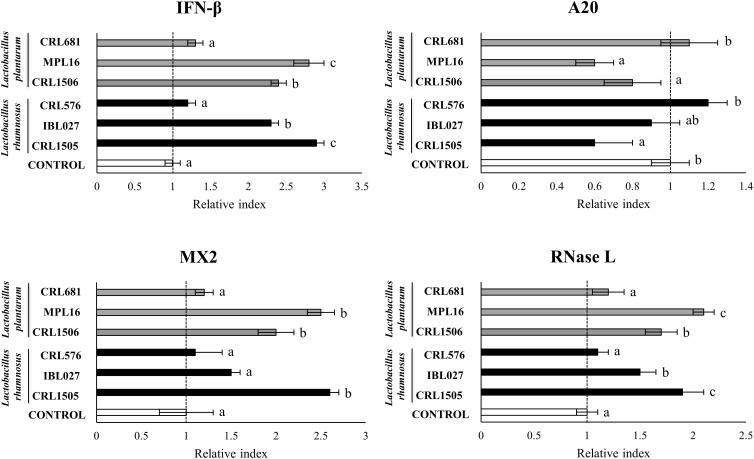
Expression of interferon (*IFN*)*-*β, antiviral factors (*MX2* and *RNASEL*), and the negative regulator *A20* genes in porcine intestinal epithelial (PIE) cells treated with *Lactobacillus rhamnosus* CRL1505, *L. rhamnosus* IBL027, *L. rhamnosus* CRL576, *Lactobacillus plantarum* CRL1506, *L. plantarum* MPL16, or *L. plantarum* CRL681 and challenged with the viral molecular–associated pattern poly(I:C), analyzed by quantitative polymerase chain reaction. Porcine intestinal epithelial cells with no lactobacilli treatment and stimulated with poly(I:C) were used as controls. The results represent data from three independent experiments. Letters indicate significant differences (*P* < 0.05), a < b < c.

Previously, we analyzed the effect of *L. rhamnosus* CRL1505 and *L. plantarum* CRL1506 on the innate immune response of PIE cells after the challenge with poly(I:C) by using a transcriptomic approach (16). From that study, we were able to select a set of potential biomarkers that would allow us to efficiently select new immunobiotic strains with antiviral capabilities including *IFN*α, *IFN*β, *RIG1*, *TLR3*, *OAS1*, *RNASEL*, *MX2*, *A20*, *CXCL5*, *CCL4*, *IL-15*, *SELL*, *SELE*, *EPCAM*, *PTGS2*, *PLA2G4A*, *PTEGES*, and *PTGER4*. Then, in order to validate this assumption, PIE cells were stimulated with different lactobacilli including immunomodulatory (*L. rhamnosus* CRL1505, *L. plantarum* CRL1506, *L. rhamnosus* IBL07, and *L. plantarum* MPL16) (8, 16, 24, 25) and non-immunomodulatory (*L. rhamnosus* CRL576 and *L. plantarum* CRL681) strains and then challenged with poly(I:C). The expression of the biomarkers was then evaluated. When the expressions of type I IFNs, antiviral factors, and the negative regulator A20 were analyzed, a strain-dependent effect was observed (Figure 1, Supplementary Figure S1). The CRL1505, CRL1506, and MPL16 strains were highly efficient for increasing *IFN*α, *MX2*, *OAS1*, and *TLR3* expression. In addition, *L. rhamnosus* CRL1505, and *L. plantarum* MPL16 were the lactobacilli with the highest capacity to increase the expression of *IFN*β and *RNASEL* (Figure 1). *Lactobacillus rhamnosus* IBL07 was capable of enhancing the mRNA levels of *IFN*α, *IFN*β, *OAS1*, *TLR3*, and *RNASEL*, but it was not as efficient as the CRL1505 and MPL16 strains. *RIG-1* was enhanced by all the immunomodulatory strains CRL1505, CRL1506, IBL027, and MPL16, whereas the non-immunomodulatory strains CRL681 and CRL576 were not able to induce changes in the expression of type I IFNs or antiviral factors (Figure 1, Supplementary Figure S1). *Lactobacillus rhamnosus* CRL1505, *L. plantarum* CRL1506, and *L. plantarum* MPL16 significantly reduced the expression of A20 in poly(I:C)-challenged PIE cells, whereas no effect was observed for the other studied strains (Figure 1).

**FIGURE 2:**
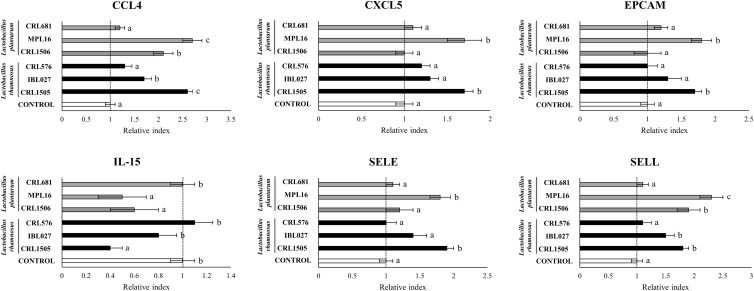
Expression of chemokines [interleukin 15 (*IL-15*), *CCL4*, and *CXCL5*] and adhesion molecules (*EPCAM*, *SELE*, and *SELL*) genes in porcine intestinal epithelial (PIE) cells treated with *Lactobacillus rhamnosus* CRL1505, *L. rhamnosus* IBL027, *L. rhamnosus* CRL576, *Lactobacillus plantarum* CRL1506, *L. plantarum* MPL16, or *L. plantarum* CRL681 and challenged with the viral molecular–associated pattern poly(I:C), analyzed by quantitative polymerase chain reaction. Porcine intestinal epithelial cells with no lactobacilli treatment and stimulated with poly(I:C) were used as controls. The results represent data from three independent experiments. Letters indicate significant differences (*P* < 0.05), a < b < c.

The CRL1505 and MPL16 strains were highly efficient in enhancing the expression of *CCL4*, *CXCL5*, *EPCAM*, and *SELE* (Figure 2). The CRL1506 and IBL027 augmented the expression of *CCL4*, but the levels of this mRNA were significantly lower than in the CRL1505 or MPL16 groups (Figure 2). The four immunomodulatory strains increased *SELL* expression, being *L. plantarum* MPL16 the most efficient to achieve this effect. The non-immunomodulatory strains CRL681 and CRL576 were not able to induce changes in the expression of chemokines and adhesion molecules (Figure 2). *Lactobacillus rhamnosus* CRL1505, *L. plantarum* CRL1506, and *L. plantarum* MPL16 significantly reduced the expression of *IL-15* in poly(I:C)-challenged PIE cells, whereas no effect was observed for the other studied strains (Figure 2).

The expressions of *PTGS2*, *PLA2G4A*, and *PTEGES* were enhanced, and *PTGER4* was reduced by the four immunomodulatory strains (Supplementary Figure S2). However, *L. rhamnosus* CRL1505 and *L. plantarum* MPL16 were more efficient than *L. plantarum* CRL1506 and *L. rhamnosus* IBL027 to increase the expression of *PTGS2*, *PLA2G4A*, and *PTEGES* and reduce *PTGER4* in poly(I:C)-challenged PIE cells. The non-immunomodulatory strains CRL681 and CRL576 were not able to induce changes in the expression of the enzymes involved in prostaglandins biosynthesis (Supplementary Figure S2).

**FIGURE 3:**
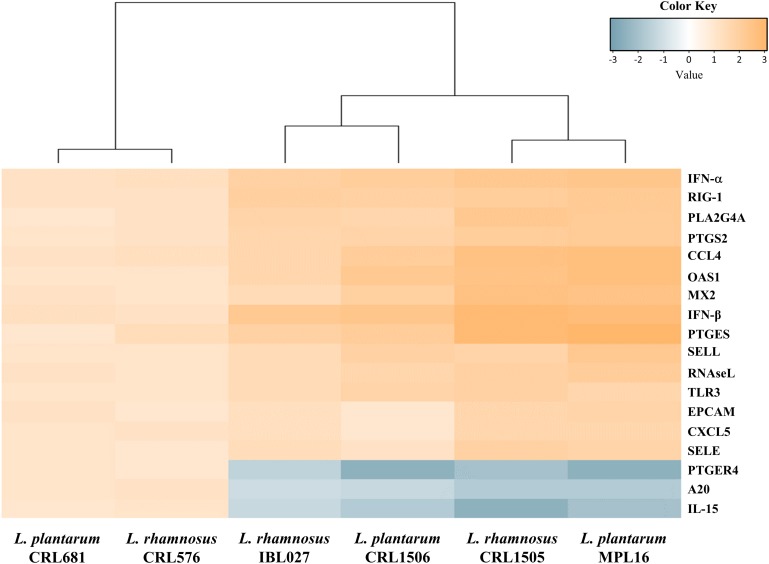
Heat map analysis of the differentially regulated genes in porcine intestinal epithelial (PIE) cells treated with *Lactobacillus rhamnosus* CRL1505, *L. rhamnosus* IBL027, *L. rhamnosus* CRL576, *Lactobacillus plantarum* CRL1506, *L. plantarum* MPL16, or *L. plantarum* CRL681 and challenged with the viral molecular–associated pattern poly(I:C).

We performed a cluster analysis to depict the transcriptomic patterns of differentially modulated genes between lactobacilli-treated and control PIE cells, in order to find the strains with similar immunomodulatory properties in the context of TLR3 activation. As shown in Figure 3, the treatments with immunomodulatory lactobacilli plus poly(I:C) clustered together and separated from the non-immunomodulatory strains. Moreover, *L. rhamnosus* CRL1505 and *L. plantarum* MPL16 were separated from *L. plantarum* CRL1506 and *L. rhamnosus* IBL027. These results indicate that *L. plantarum* MPL16 would have the ability to differentially regulate the immunotranscriptomic response in poy(I:C)-challenged PIE cells (Supplementary Figure S3) in a way comparable to that previously reported for *L. rhamnosus* CRL1505 (16, 20).

### Modulation of TLR3-Triggered Intestinal Immune Response in Mice by Lactobacilli

Next, we were interested in finding out whether the differences in the immunomodulatory activities of CRL1505, MPL16, CRL1506, and IBL027 observed in PIE cells could be also found in an *in vivo* model. Then, lactobacilli were given orally to different groups of mice, and the intestinal cytokine profile was evaluated before (basal levels) and after the intraperitoneal challenge with poly(I:C). As observed in Supplementary Figure S4, a strain-dependent ability in the modulation of the basal levels of intestinal cytokines was detected. The four immunomodulatory strains increased the intestinal levels of IFN-β, IFN-γ, and IL-10; however, *L. rhamnosus* CRL1505 and *L. plantarum* MPL16 were more efficient than the other strains to augment IFN-γ and IL-10. The non-immunomodulatory strains CRL681 and CRL576 did not modify the levels of intestinal IFN-β. Interestingly, *L. plantarum* CRL681 increased the levels of intestinal IFN-γ and IL-10 (Supplementary Figure S4). All the lactobacilli strains with the exception of *L. rhamnosus* CRL576 increased TNF-α, whereas the CRL1505, MPL16, IBL027, and CRL681 enhanced the intestinal levels of IL-6 (Supplementary Figure S4). The basal concentration of IL-15 was under the detection limits in all the experimental groups (data not shown).

**FIGURE 4:**
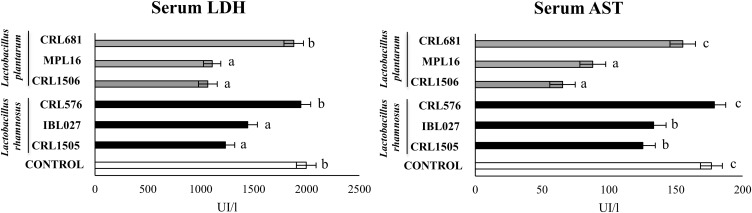
Levels of serum injury markers [lactate dehydrogenase (LDH) and aspartate aminotransferase (AST)] in mice orally treated with *Lactobacillus rhamnosus* CRL1505, *L. rhamnosus* IBL027, *L. rhamnosus* CRL576, *Lactobacillus plantarum* CRL1506, *L. plantarum* MPL16, or *L. plantarum* CRL681 (10^8^ cells/mouse per day for five consecutive days) and then challenged by the intraperitoneal route with 30 μg of the viral molecular–associated pattern poly(I:C). Mice with no lactobacilli treatment and challenged with poly(I:C) were used as controls. Serum injury markers were evaluated on day 2 after poly(:C) administration. The results represent data from three independent experiments (*n* = 6 per group). Letters indicate significant differences (*P* < 0.05), a < b < c.

We evaluated the biochemical markers LDH and AST in order to study the inflammatory damage after poly(I:C) administration (Figure 4). As we reported previously (8), the intraperitoneal challenge with poly(I:C) significantly increased LDH and AST activities in serum samples. The four immunomodulatory strains decreased serum LDH and AST, whereas the non-immunomodulatory strains CRL681 and CRL576 did not modify the levels of those markers (Figure 4).

**FIGURE 5:**
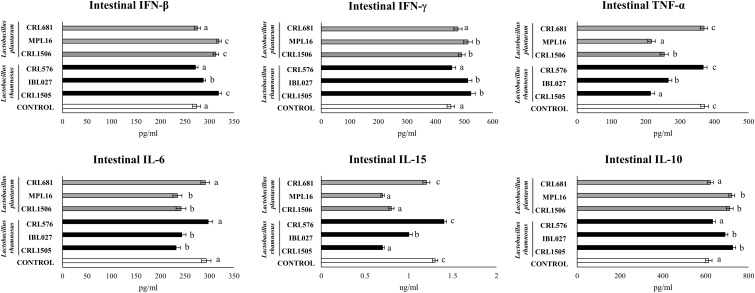
Levels of intestinal interferons (IFN-β and IFN-γ), proinflammatory cytokines [TNF-α and interleukin 6 (IL-6)] and IL-10 in mice orally treated with *Lactobacillus rhamnosus* CRL1505, *L. rhamnosus* IBL027, *L. rhamnosus* CRL576, *Lactobacillus plantarum* CRL1506, *L. plantarum* MPL16, or *L. plantarum* CRL681 (10^8^ cells/mouse per day for 5 consecutive days) and then challenged by the intraperitoneal route with 30 μg of the viral molecular–associated pattern poly(I:C). Mice with no lactobacilli treatment and challenged with poly(I:C) were used as controls. Immune factors were evaluated on day 2 after poly(:C) administration. The results represent data from three independent experiments (*n* = 6 per group). Letters indicate significant differences (*P* < 0.05), a < b < c.

In addition, the intraperitoneal administration of poly(I:C) significantly increased the levels of IFN-β; IFN-γ; the proinflammatory cytokines TNF-α, IL-6, and IL-15; and the regulatory cytokine IL-10 in the intestinal fluid (Figure 5). The four immunomodulatory strains were able to enhance the intestinal levels of IFN-β and IFN-γ after TLR3 activation. The CRL1505, MPL16, CRL1506, and IBL027 strain were also capable of significantly reducing the concentrations of TNF-α, IL-6, and IL-15 and increasing the levels of IL-10 in the intestine when compared to control mice (Figure 5). Of note, *L. rhamnosus* CRL1505 and *L. plantarum* MPL16 were more efficient than the other strains to reduce TNF-α and IL-15. The non-immunomodulatory strains CRL681 and CRL576 did not modify the levels of IFN-β, IFN-γ, TNF-α, IL-6, IL-15, or IL-10 when compared to controls (Figure 5).

**FIGURE 6:**
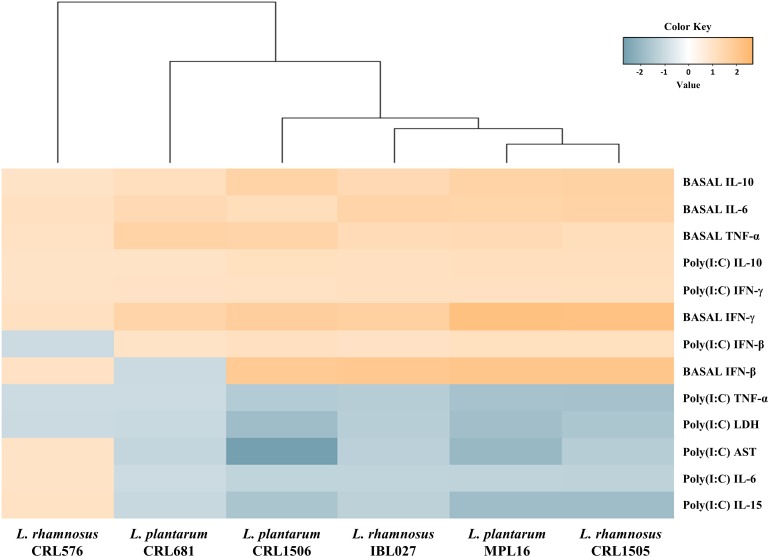
Heat map analysis of the differentially regulated intestinal immune factors and serum injury markers before (basal) and after poly(I:C) challenge. Mice were orally treated with *Lactobacillus rhamnosus* CRL1505, *L. rhamnosus* IBL027, *L. rhamnosus* CRL576, *Lactobacillus plantarum* CRL1506, *L. plantarum* MPL16, or *L. plantarum* CRL681, and then challenged by the intraperitoneal route with the viral molecular–associated pattern poly(I:C).

Again, we performed a cluster analysis to depict the differentially modulated cytokines and injury markers between lactobacilli-treated and control mice, in order to find the strains with similar immunomodulatory properties in the *in vivo* mice model. As shown in Figure 6, a clear strain-dependent effect was observed in the ability of lactobacilli to modulate cytokines before and after poly(I:C) challenge. Interestingly, *L. rhamnosus* CRL1505 and *L. plantarum* MPL16 clustered together and separated from the other immunomodulatory strains *L. plantarum* CRL1506 and *L. rhamnosus* IBL027. These results allow us to speculate that *L. plantarum* MPL16 would have the ability to differentially regulate the intestinal innate immune response in poy(I:C)-challenged mice and protect against the inflammatory damage (Supplementary Figure S5) in a way comparable to that previously reported for *L. rhamnosus* CRL1505 (8).

### Modulation of TLR3-Triggered Respiratory Immune Response in Mice by Lactobacilli

We have previously reported that orally administered *L. rhamnosus* CRL1505 is able to beneficially modulate the respiratory immune response triggered by TLR3 activation (7) and improve the resistance against influenza virus (10) and RSV (9). We also showed that the capacity of orally administered CRL1505 strain to modulate immunity in distal mucosal sites is not sheared by the immunomodulatory strain *L. plantarum* CRL1506. Then, taking into considerations the similarities found in this work between the CRL1505 and MPL16 strains, we were interested in finding out whether orally administered *L. plantarum* MPL16 was able to influence respiratory tract immunity. For this purpose, lactobacilli were given orally to different groups of mice, and the serum and respiratory cytokine profile was evaluated before (basal levels) and after the nasal challenge with poly(I:C). The changes in the profile of cytokines induced in the BAL (Supplementary Figure S6) and serum (Supplementary Figure S7) by lactobacilli indicated a clear strain-dependent effect. When the levels of the different cytokines were analyzed in BAL, it was shown that only *L. rhamnosus* CRL1505 and *L. plantarum* MPL16 enhanced the concentrations of IFN-β. In addition, CRL1505, MPL16 and IBL027 increased the basal levels of IFN-γ and IL-10. However, *L. rhamnosus* IBL027 was less efficient than the CRL1505 and MPL16 to induce the up-regulation of those cytokines (Supplementary Figure S6). The basal levels of respiratory TNF-α were increased by *L. rhamnosus* CRL1505, *L. plantarum* MPL16, and *L. plantarum* CRL1506, whereas the CRL681 and CRL576 treatments induced no changes in any of the cytokines evaluated in the respiratory tract (Supplementary Figure S6).

All the immunomodulatory strains were capable of enhancing the concentrations of IFN-β, IFN-γ, TNF-α, and IL-10 in serum when compared to controls. However, *L. rhamnosus* CRL1505 and *L. plantarum* MPL16 were more efficient than the other immunomodulatory strains to increase the basal levels of serum IFN-γ, TNF-α, and IL-10. *Lactobacillus rhamnosus* CRL576 and *L. plantarum* CRL681 did not induce significant changes in serum cytokines when compared to controls (Supplementary Figure S7).

**FIGURE 7:**
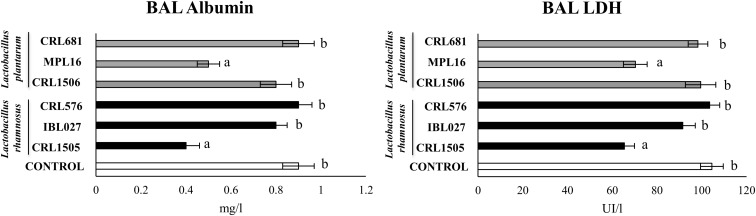
Levels of bronchoalveolar lavage (BAL) injury markers [albumin and lactate dehydrogenase (LDH)] in mice orally treated with *Lactobacillus rhamnosus* CRL1505, *L. rhamnosus* IBL027, *L. rhamnosus* CRL576, *Lactobacillus plantarum* CRL1506, *L. plantarum* MPL16, or *L. plantarum* CRL681 (10^8^ cells/mouse per day for five consecutive days) and then challenged by the nasal route with 250 μg of the viral molecular–associated pattern poly(I:C) for 3 consecutive days. Mice with no lactobacilli treatment and challenged with poly(I:C) were used as controls. Bronchoalveolar lavage injury markers were evaluated on day 2 after the last poly(:C) administration. The results represent data from three independent experiments (*n* = 6 per group). Letters indicate significant differences (*P* < 0.05), a < b < c.

We also evaluated the levels of the biochemical markers albumin and LDH in BAL as indicators of lung injury (7, 9, 10). The nasal challenge of mice with poly(I:C) significantly altered lungs function and induced lung injuries as demonstrated by the increased levels of BAL albumin and LDH (Figure 7), reflecting alteration of the alveolar–capillary barrier and local cellular damage. Only *L. rhamnosus* CRL1505 and *L. plantarum* MPL16 treatments were able to significantly reduce the levels of BAL LDH and albumin when compared to controls (Figure 7).

**FIGURE 8:**
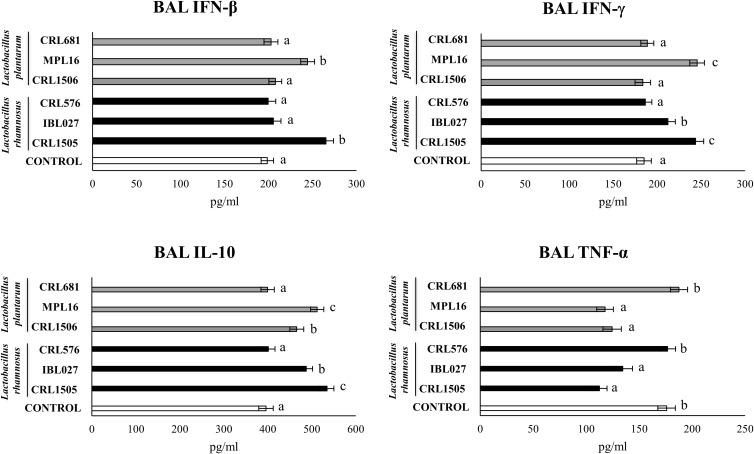
Levels of bronchoalveolar lavage (BAL) interferons (IFN-β and IFN-γ), TNF-α, and interleukin 10 (IL-10) in mice orally treated with *Lactobacillus rhamnosus* CRL1505, *L. rhamnosus* IBL027, *L. rhamnosus* CRL576, *Lactobacillus plantarum* CRL1506, *L. plantarum* MPL16, or *L. plantarum* CRL681 (10^8^ cells/mouse per day for 5 consecutive days) and then challenged by the nasal route with 250 μg of the viral molecular–associated pattern poly(I:C) for three consecutive days. Mice with no lactobacilli treatment and challenged with poly(I:C) were used as controls. Bronchoalveolar lavage immune factors were evaluated on day 2 after the last poly(:C) administration. The results represent data from three independent experiments (*n* = 6 per group). Letters indicate significant differences (*P* < 0.05), a < b < c.

The nasal administration poly(I:C) significantly increased serum (Supplementary Figure S8) and respiratory (Figure 8) levels of IFN-β, IFN-γ, TNF-α, and IL-10. *Lactobacillus rhamnosus* CRL1505 and *L. plantarum* MPL16 enhanced the levels of IFN-β and IFN-γ in both serum and BAL, whereas *L. rhamnosus* IBL027 was capable of increasing only BAL IFN-γ. The four immunomodulatory strains were capable of reducing serum and BAL levels of TNF-α, however; the CRL1505 and MPL16 strains were more effective to down-regulate this inflammatory cytokine in the respiratory tract (Figure 8). In addition, the four immunomodulatory strains increased IL-10 in serum (Supplementary Figure S8), but only *L. rhamnosus* CRL1505 and *L. plantarum* MPL16 enhanced the levels of this immunoregulatory cytokine in the respiratory tract (Figure 8).

**FIGURE 9:**
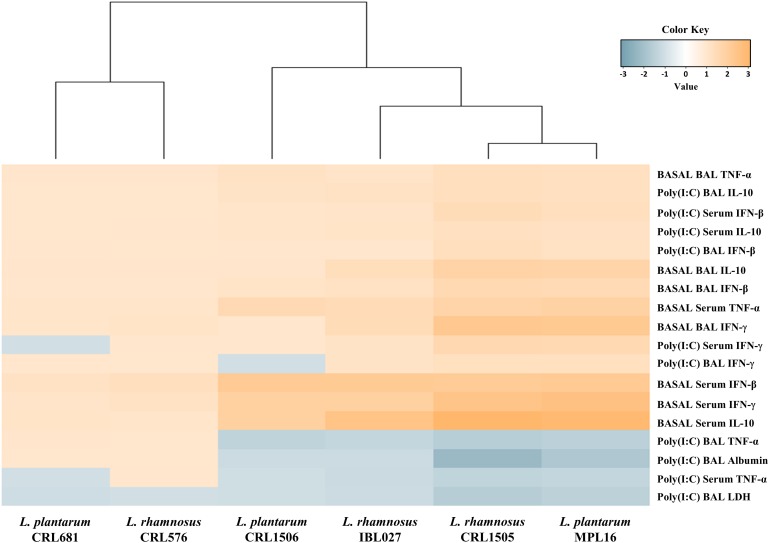
Heat map analysis of the differentially regulated serum and bronchoalveolar lavage (BAL) immune factors and BAL injury markers before (basal) and after poly(I:C) challenge. Mice were orally treated with *Lactobacillus rhamnosus* CRL1505, *L. rhamnosus* IBL027, *L. rhamnosus* CRL576, *Lactobacillus plantarum* CRL1506, *L. plantarum* MPL16, or *L. plantarum* CRL681 and then challenged by the nasal route with the viral molecular–associated pattern poly(I:C) for three consecutive days.

We performed a cluster analysis to depict the differentially modulated serum and BAL cytokines and lung injury markers between lactobacilli-treated and control mice. As shown in Figure 9, a clear strain-dependent effect was observed in the ability of lactobacilli to modulate immunity in the respiratory tract. Of note, *L. rhamnosus* CRL1505 and *L. plantarum* MPL16 clustered together and separated from the other immunomodulatory strains. These results allow us to speculate that *L. plantarum* MPL16 would have the ability to differentially regulate the systemic and respiratory innate immune response in poy(I:C)-challenged mice and protect against the lung inflammatory damage (Supplementary Figure S9) in a way comparable to that previously reported for *L. rhamnosus* CRL1505 (7).

### Enhancement of the Resistance Against RSV Infection by *L. plantarum* MPL16

**FIGURE 10:**
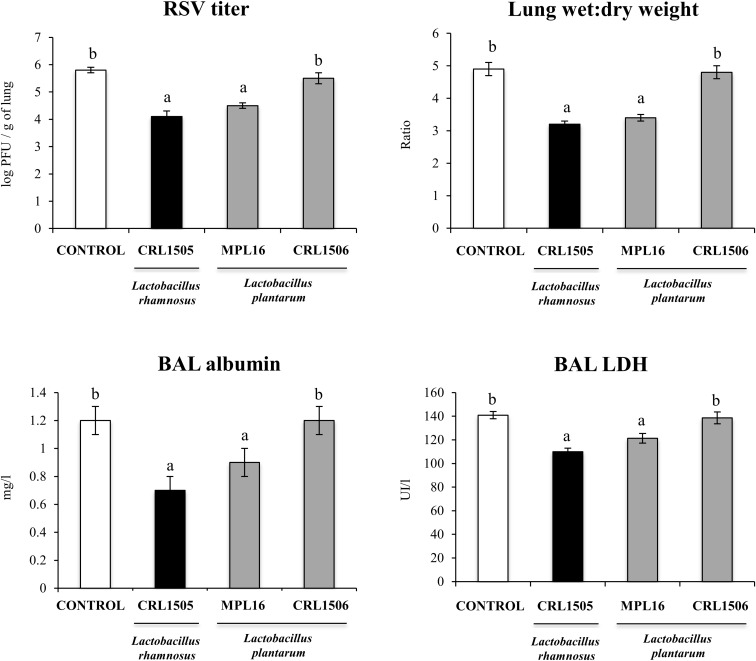
Lung respiratory syncytial virus (RSV) titers, lung wet-to-dry ratio, and levels of bronchoalveolar lavage (BAL) injury markers [albumin and lactate dehydrogenase (LDH)] in mice orally treated with *Lactobacillus rhamnosus* CRL1505, *Lactobacillus plantarum* CRL1506, or *L. plantarum* MPL16 (10^8^ cells/mouse per day for five consecutive days) and then challenged by the nasal route with RSV. Mice with no lactobacilli treatment and infected with RSV were used as controls. Respiratory syncytial virus titers and injury markers were evaluated on day 2 after viral infection. The results represent data from three independent experiments (*n* = 6 per group). Letters indicate significant differences (*P* < 0.05), a < b < c.

**FIGURE 11:**
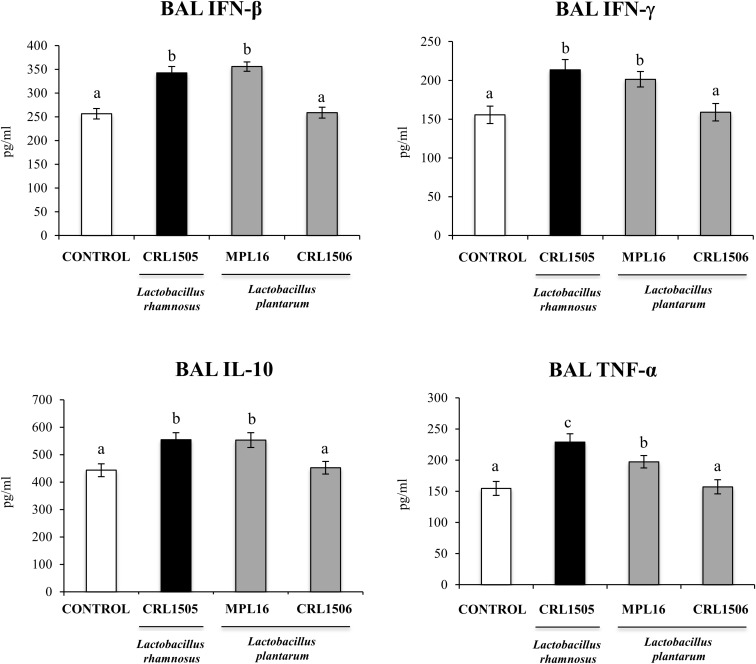
Levels of bronchoalveolar lavage (BAL) interferons (IFN-β and IFN-γ), TNF-α, and interleukin 10 (IL-10) in mice orally treated with *Lactobacillus rhamnosus* CRL1505, *Lactobacillus plantarum* CRL1506, or *L. plantarum* MPL16 (10^8^ cells/mouse per day for five consecutive days) and then challenged by the nasal route with RSV. Mice with no lactobacilli treatment and infected with RSV were used as controls. Immune markers were evaluated on day 2 after viral infection. The results represent data from three independent experiments (*n* = 6 per group). Letters indicate significant differences (*P* < 0.05), a < b < c.

Finally, we aimed to test whether the oral administration of *L. plantarum* MPL16 was able to confer protection against a respiratory virus challenge. For this purpose, mice were fed the MPL16 strain and then nasally challenged with RSV. *Lactobacillus rhamnosus* CRL1505 and *L. plantarum* CRL1506 were used as positive and negative controls, respectively, according to our previous work demonstrating their different ability to protect against this viral pathogen (9). As shown in Figure 10, both *L. rhamnosus* CRL1505 and *L. plantarum* MPL16 were equally effective in reducing RSV lung titers, whereas the CRL1506 strain did not induce changes when compared to control mice. In addition, the MPL16 and CRL1505 strains significantly reduced the levels of the markers of lung damage, whereas *L. plantarum* CRL1506 was not able to achieve this effect (Figure 10). The levels of respiratory IFN-β, IFN-γ, TNF-α, and IL-10 were also evaluated after the challenge with RSV (Figure 11). Both *L. rhamnosus* CRL1505 and *L. plantarum* MPL16 enhanced the levels of IFN-β, IFN-γ, and IL-10 in BAL, whereas *L. plantarum* CRL1506 was not capable of increasing these cytokines when compared to controls. The CRL1506 strain did not induce changes in the levels of BAL TNF-α when compared to the control group. The MPL16 and CRL1505 strains significantly increased the respiratory levels of TNF-α, being *L. rhamnosus* CRL1505 more effective than *L. plantarum* MPL16 to achieve this effect (Figure 11).

## Discussion

In middle- and low-income countries, intestinal and respiratory viral infections are the most common and deadly diseases in children (26–28). The use of functional foods such as those containing immunomodulatory probiotic lactobacilli has been proposed to stimulate the intestinal and the respiratory immune system, improving the outcome of viral diarrhea and pneumonia simultaneously. An example of the high probability of success of this strategy is the “Yogurito Nutritional Program” implemented in Argentina (2, 29). The program uses a probiotic yogurt containing the immunobiotic strain *L. rhamnosus* CRL1505 to prevent respiratory and gastrointestinal diseases by enhancing the immunological system of children attending public schools. Then, the selection of new immunobiotic strains that have different biotechnological properties but the same or better immunomodulatory capacities than *L. rhamnosus* CRL1505 could enhance the development of various types of functional foods that could be used around the world to reduce the incidence and severity of intestinal and pulmonary infections caused by viruses. Such selection, in addition to being efficient, should be performed using *in vitro* systems in order to minimize the use of experimental animals.

In this work, we demonstrated that the study of biomarkers expression in poly(I:C)-challenged PIE cells is a very effective tool for the selection of immunobiotics with the ability to modulate intestinal and respiratory antiviral immunity. The transcriptional profiling performed *in vitro* in poly(I:C)-challenged PIE cells in this work allowed us to select a new immunobiotic strain, *L. plantarum* MPL16, with the ability to stimulate *in vivo* the local (intestinal) and distal (respiratory) mucosal immune systems.

*Lactobacillus rhamnosus* CRL1505 and *L. plantarum* MPL16 similarly modulated gene expression in poly(I:C)-challenged PIE cells inducing significant increases of both *IFN-*α and *IFN-*β and in the antiviral factors *MX2*, *OAS1*, and *RNASEL*. Our previous studies in PIE cells showed that rotavirus can be detected by this cell line through TLR3, inducing the expression of *IFN-*β and up-regulating the antiviral genes *MxA* and *RNASEL* (15). Moreover, those studies demonstrated that immunobiotics strains with the ability to enhance *IFN-*β, *MxA*, and *RNASEL* were also capable of reducing rotavirus replication. We demonstrated that immunobiotic strains such as *L. rhamnosus* CRL1505 (16) and *Bifidobacterium infantis* MCC12 (15) increased the expression of IFN-β and antiviral factors by reducing the expression of *A20*. The down-regulation of *A20* gene expression in poly(I:C)-challenged PIE cells results in the improved activation of IRF3 and NF-κB signaling pathways, which increase the expression of not only *IFN-*α, *IFN-*β, *MX2*, *OAS1*, and *RNASEL* but in addition several other antiviral factors including *OASL*, *MX1*, *OAS2*, *RNASE4, IFIT1*, *IFIT3*, *IFIT2*, and *IFIT5* (Supplementary Figure S3) (16). The *in vivo* experiments in mice performed here also demonstrated that *L. plantarum* MPL16 is able to enhance the intestinal levels of IFN-β. Moreover, MPL16-treated mice also showed higher levels of intestinal IFN-γ as observed by *L. rhamnosus* CRL1505. It was reported that infection with rotavirus in suckling mice induces a significant up-regulation of different types of IFNs in the intestine including IFN-γ by T cells and type I IFNs by dendritic cells (DCs) and IECs (30). Interferons binding to their cognate cell surface receptors activate positive feedback loops that amplify the expression of IFNs as well as more than 300 different IFN-stimulated genes (31). This IFNs release then efficiently amplify the expression of antiviral proteins targeting a variety of viral replication steps in uninfected bystander cells. It was also shown in *in vitro* studies that the addition of purified exogenous IFNs after rotavirus infection of human IECs does not significantly hamper viral replication; however, IFN treatment of cells prior to viral challenge is required to achieve an efficient restriction of rotavirus replication (32).

The excessive activation of the inflammatory response or the failure in the mechanisms that control it significantly contributes to the injury of the infected tissue during viral infections. Intestinal epithelial cells produce a variety of cytokines and chemokines in response to the viral attack, including IL-6, IL-8, TNF-α, and granulocyte-macrophage colony-stimulating factor. The production of those inflammatory factors is important for the protection against the viral infection through their direct antiviral effects (33) or the recruitment and activation of phagocytes (34). However, infiltration of immune cells to the intestinal mucosa can contribute to the local damage mediated by the inflammatory and oxidative stress (35). In addition, it was reported that purified dsRNA from rotavirus is able to induce severe intestinal damage in mice through the activation of TLR3 signaling pathway (36). Moreover, it was also demonstrated that the intraperitoneal administration of the synthetic dsRNA poly(I:C) to mice mimics the inflammatory intestinal immune response elicited by rotavirus and induce mucosal erosion, villous atrophy, intestinal wall attenuation, and diarrhea (8, 36, 37). Interestingly, it was shown that the intestinal damage triggered by dsRNA-TRL3 interaction is mediated by the increased expression of IL-15 and retinoic acid early inducible 1 (RAE1) in IECs, which induce the activation of CD3^+^NK1.1^+^CD8αα^+^ intraepithelial lymphocytes (IELs) and promote epithelial destruction through the RAE1–NKG2D interaction (Supplementary Figure S5) (38). We reported previously that *L. rhamnosus* CRL1505 is able to significantly reduce the expression of *IL-15* and *RAE1* in poly(I:C)-challenged PIE cells (16) and to reduce the levels of intestinal TNF-α and IL-15 and diminish the gut damage mediated by CD3^+^NK1.1^+^CD8αα^+^ IELs in mice after TLR3 activation (8). Here, we found that *L. plantarum* MPL16 is able to modulate the cytokine profile expression triggered by TLR3 activation in PIE cells as well as in mice intestinal mucosa, in a way similar to that observed for the CRL1505 strain. Then, it is tempting to speculate that *L. plantarum* MPL16 would have the ability to beneficially regulate intestinal inflammation in the context of TLR3 activation; however, more detailed studies using viral challenges are necessary to demonstrate this effect.

Our results allow us to speculate that *L. plantarum* MPL16 would be capable to modulate IECs innate immune response, improve the resistance to rotavirus infection, and reduce the severity of inflammatory-mediated damage, as we have previously demonstrated *in vitro* (16, 20), *in vivo* (8), and in clinical trials (2, 29) for the CRL1505 strain.

We also demonstrated here that orally administered *L. plantarum* MPL16 is able to differentially modulate the respiratory antiviral immune response. We reported previously that the improvement of IFN-β production by CD11c^+^SiglecF^+^ alveolar macrophages and IFN-γ by CD3^+^CD4^+^ T cells is related to the ability of immunobiotic treatments to enhance resistance to respiratory virus (39, 40), in line with studies demonstrating that these immune cell populations are the main producer of IFNs during pulmonary viral infections (36, 37). The increased levels of respiratory IFN-γ and IFN-β found in *L. plantarum* MPL16–treated mice correlated with the improved resistance of mice to RSV infection. On the other hand, we have extensively used a mice experimental model of lung inflammation based on the nasal administration of poly(I:C) in order to mimic the respiratory innate antiviral immune response triggered by RSV and to evaluate the beneficial effects of immunobiotic bacteria (7, 9, 10, 39, 40). The respiratory priming with the TLR3 agonist induces a marked inflammatory damage characterized by impaired alveolar–capillary barrier function and epithelial cell death as well as increased levels of TNF-α, IL-6, IL-8, and MCP-1. Prominent improvements of the IL-8, MIP-1, RANTES, MCP-1, TNF-α, and IL-6 have been reported in both experimentally RSV-infected mice and naturally RSV-infected children (41). The increase in the respiratory levels of those inflammatory factors, especially TNF-α, contributes to clearance of the virus during the early stages of RSV infection; however, their continued production exacerbates lung injuries during the late stages of infection (41, 42). Then, the appropriate regulation of the respiratory immune response is also essential for the protection of RSV-infected hosts. In this regard, it was demonstrated that IL-10 has a crucial role in regulating the severity of RSV infection (42, 43). The deficiency of IL-10 does not affect RSV load in lungs, but significantly enhances the inflammatory cells influx into the lung, promotes lung damage, and increases weight loss of infected mice (42, 43). The results of this work demonstrated that orally administered *L. plantarum* MPL16 is able to reduce the levels of inflammatory factors, increase IL-10, and significantly diminish the markers of lung tissue damage after the nasal administration of poly(I:C). Moreover, we also demonstrated here that orally administered *L. plantarum* MPL16 significantly reduced the respiratory injury markers after RSV challenge and differentially modulated the levels of respiratory proinflammatory and anti-inflammatory cytokines induced by the viral infection. Those effects were similar to the previously described for the CRL1505 strain (Supplementary Figure S9) (7, 9, 10, 39).

The results of this work confirm that new immunobiotics strains with the ability of stimulating both local and distal antiviral immune responses when orally administered can be efficiently selected by evaluating the expression of appropriate biomarkers of the transcriptomic profile of poly(I:C)-challenged PIE cells. The comparison of the transcriptomic patterns of differentially modulated genes in PIE cells treated with different lactobacilli allowed us to select the MPL16 strain that clustered together with the immunobiotic strain *L. rhamnosus* CRL1505, which has been proved to differentially modulate the intestinal and the respiratory antiviral responses and protect against enteric and respiratory viruses (2, 7, 9, 10). The *in vivo* studies performed here conclusively demonstrated that *L. plantarum* MPL16 modulated the profiles of intestinal, serum, and respiratory cytokines; reduced the inflammatory damage triggered by TLR3 activation in both the intestinal and respiratory mucosa; and improved the resistance to RSV infection. The immunological changes induced by *L. plantarum* MPL16 were not different from those previously reported for the CRL1505 strain. Further mechanistic studies evaluating comparatively the effects of CRL1505 and MPL16 strains in different immune cell populations such as DCs and macrophages, as well as more detailed molecular and genomic characterization of both lactobacilli strains, could contribute significantly to the understanding of the molecular mechanisms involved in the ability of immunobiotics to stimulate distant mucosal sites such as the respiratory tract.

Of note, the biotechnological properties of the two strains are different. Whereas *L. rhamnosus* CRL1505 has been used mainly in the development of dairy functional products (2, 44), the MPL16 strain has shown a remarkable ability to growth and ferment wakame (*Undaria pinnatifida*) that is the most popular and economically important edible brown algae in Asian countries (24, 45). Then, the different biotechnological properties of *L. plantarum* MPL16 could potentiate the development of non-dairy functional foods or feeds with the ability to improve antiviral immunity in the intestine and the respiratory tract.

## Data Availability Statement

The datasets generated for this study are available on request to the corresponding author.

## Ethics Statement

The animal study was reviewed and approved by the Ethical Committee of Animal Care CERELA-CONICET, Tucuman, Argentina.

## Author Contributions

JV and HK designed the study and manuscript writing. LA, VG-C, YI, and MI did the laboratory work. LA, MI, and YS did the statistical analysis. AG-C, HA, HT, JV, and HK contributed to data analysis and interpretation. All authors read and approved the manuscript.

## Conflict of Interest

The authors declare that the research was conducted in the absence of any commercial or financial relationships that could be construed as a potential conflict of interest.

## Supplementary Material

FIGURE S1 Expression of interferon (*IFN*)*-*α, and antiviral factors (*TLR3, RIG-1* and *OAS1*) genes in porcine intestinal epithelial (PIE) cells treated with *Lactobacillus rhamnosus* CRL1505, *L. rhamnosus* IBL027, *L. rhamnosus* CRL576, *Lactobacillus plantarum*C RL1506, *L. plantarum* MPL16, or *L. plantarum* CRL681 and challenged with the viral molecular associated pattern poly(I:C), analyzed by qPCR. PIE cells with no lactobacilli treatment and stimulated with poly(I:C) were used as controls. The results represent data from three independent experiments. Letters indicate significant differences (*P* < 0.05), a < b < c.

FIGURE S2 Expression of genes involved in prostaglandins biosynthesis (*PTGS2, PLA2G4A, PTGER4* and *PTGES*) in porcine intestinal epithelial (PIE) cells treated with *Lactobacillus rhamnosus* CRL1505, *L. rhamnosus* IBL027, *L. rhamnosus* CRL576, *Lactobacillus plantarum* CRL1506, *L. plantarum* MPL16, or *L. plantarum* CRL681 and challenged with the viral molecular associated pattern poly(I:C), analyzed by qPCR. PIE cells with no lactobacilli treatment and stimulated with poly(I:C) were used as controls. The results represent data from three independent experiments. Letters indicate significant differences (*P* < 0.05), a < b < c.

FIGURE S3 Global overview of the signaling pathways and immune genes differentially regulated in PIE cells treated with *L. rhamnosus* CRL1505 or *L. plantarum* MPL16 and challenged with poly(I:C). Detailed immunomodulatory mechanisms were previously studied for *L. rhamnosus* CRL1505 (16, 20).

FIGURE S4 Levels of intestinal interferons (IFN-β and IFN-γ), proinflammatory cytokines (TNF-α and IL-6) and IL-10 in mice orally treated with *Lactobacillus rhamnosus* CRL1505, *L. rhamnosus* IBL027, *L. rhamnosus* CRL576, *Lactobacillus plantarum* CRL1506, *L. plantarum* MPL16, or *L. plantarum* CRL681 (10^8^ cells/mouse/day for five consecutive days). Mice with no lactobacilli treatment were used as controls. Immune factors were evaluated on day 6. The results represent data from three independent experiments (*n* = 6 per group). Letters indicate significant differences (*P* < 0.05), a < b < c.

FIGURE S5 Global overview of the effect of *L. rhamnosus* CRL1505 and *L. plantarum* MPL16 on the intestinal inflammatory injury induced by poly(I:C) and mediated by the activation of CD3^+^NK1.1^+^CD8αα^+^ intraepithelial lymphocytes (IELs) that promote epithelial destruction through the RAE1–NKG2D interaction. Detailed immunomodulatory mechanisms were previously studied for *L. rhamnosus* CRL1505 (8).

FIGURE S6 Levels of bronco-alveolar lavages (BAL) interferons (IFN-β and IFN-γ), TNF-α and IL-10 in mice orally treated with *Lactobacillus rhamnosus* CRL1505, *L. rhamnosus* IBL027, *L. rhamnosus* CRL576, *Lactobacillus plantarum* CRL1506, *L. plantarum* MPL16, or *L. plantarum* CRL681 (10^8^ cells/mouse/day for five consecutive days). Mice with no lactobacilli treatment were used as controls. Immune factors were evaluated on day 6. The results represent data from three independent experiments (*n* = 6 per group). Letters indicate significant differences (*P* < 0.05), a < b < c.

FIGURE S7 Levels of serum interferons (IFN-β and IFN-γ), TNF-α and IL-10 in mice orally treated with *Lactobacillus rhamnosus* CRL1505, *L. rhamnosus* IBL027, *L. rhamnosus* CRL576, *Lactobacillus plantarum* CRL1506, *L. plantarum* MPL16, or *L. plantarum* CRL681 (10^8^ cells/mouse/day for five consecutive days). Mice with no lactobacilli treatment were used as controls. Immune factors were evaluated on day 6. The results represent data from three independent experiments (*n* = 6 per group). Letters indicate significant differences (*P* < 0.05), a < b < c.

FIGURE S8 Levels of serum interferons (IFN-β and IFN-γ), TNF-α and IL-10 in mice orally treated with *Lactobacillus rhamnosus* CRL1505, *L. rhamnosus* IBL027, *L. rhamnosus* CRL576, *Lactobacillus plantarum* CRL1506, *L. plantarum* MPL16, or *L. plantarum* CRL681 (10^8^ cells/mouse/day for five consecutive days), and then challenged by the nasal route with 250 μg of the viral molecular associated pattern poly(I:C) for three consecutive days. Mice with no lactobacilli treatment and challenged with poly(I:C) were used as controls. Serum immune factors were evaluated on day 2 after the last poly(:C) administration. The results represent data from three independent experiments (*n* = 6 per group). Letters indicate significant differences (*P* < 0.05), a < b < c.

FIGURE S9 Global overview of the effect of *L. rhamnosus* CRL1505 and *L. plantarum* MPL16 on the respiratory innate immune response triggered by poly(I:C). Detailed immunomodulatory mechanisms were previously studied for *L. rhamnosus* CRL1505 (7, 9).
