# Supplementary figures and images for: Efficient Selection of New Immunobiotic Strains With Antiviral Effects in Local and Distal Mucosal Sites by Using Porcine Intestinal Epitheliocytes
Source: Front Immunol. 2020 Apr 8;11:543. doi: 10.3389/fimmu.2020.00543 (PMC7156603; doi:10.3389/fimmu.2020.00543)

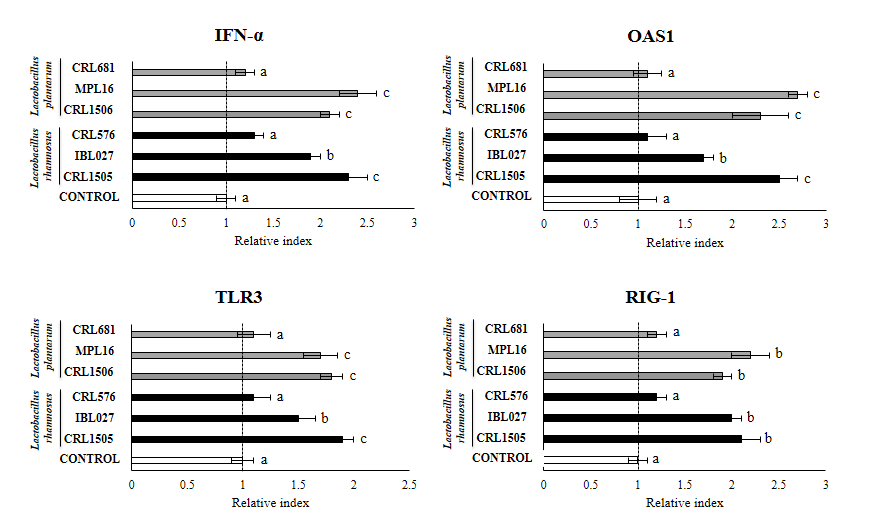

Supplement: FIGURE S1 — Expression of interferon (IFN)-α, and antiviral factors (TLR3, RIG-1 and OAS1) genes in porcine intestinal epithelial (PIE) cells treated with Lactobacillus rhamnosus CRL1505, L. rhamnosus IBL027, L. rhamnosus CRL576, Lactobacillus plantarumC RL1506, L. plantarum MPL16, or L. plantarum CRL681 and challenged with the viral molecular associated pattern poly(I:C), analyzed by qPCR. PIE cells with no lactobacilli treatment and stimulated with poly(I:C) were used as controls. The results represent data from three independent experiments. Letters indicate significant differences (P < 0.05), a < b < c. [file Image_1.TIF]

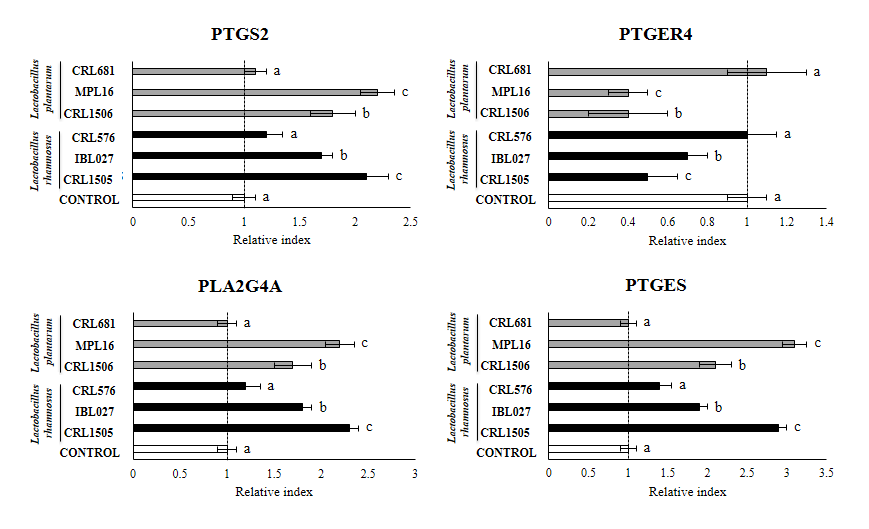

Supplement: FIGURE S2 — Expression of genes involved in prostaglandins biosynthesis (PTGS2, PLA2G4A, PTGER4 and PTGES) in porcine intestinal epithelial (PIE) cells treated with Lactobacillus rhamnosus CRL1505, L. rhamnosus IBL027, L. rhamnosus CRL576, Lactobacillus plantarum CRL1506, L. plantarum MPL16, or L. plantarum CRL681 and challenged with the viral molecular associated pattern poly(I:C), analyzed by qPCR. PIE cells with no lactobacilli treatment and stimulated with poly(I:C) were used as controls. The results represent data from three independent experiments. Letters indicate significant differences (P < 0.05), a < b < c. [file Image_2.TIF]

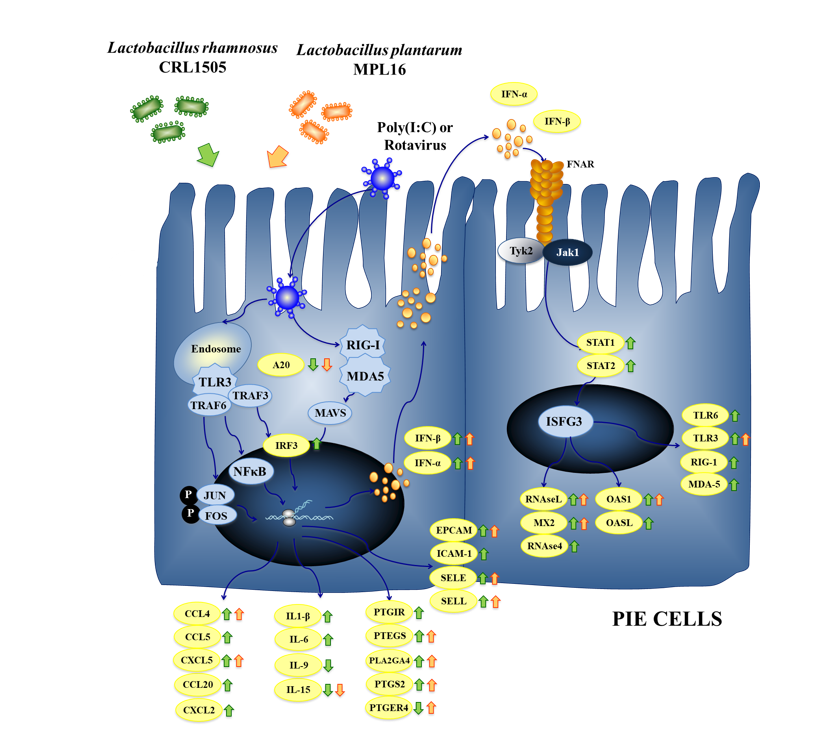

Supplement: FIGURE S3 — Global overview of the signaling pathways and immune genes differentially regulated in PIE cells treated with L. rhamnosus CRL1505 or L. plantarum MPL16 and challenged with poly(I:C). Detailed immunomodulatory mechanisms were previously studied for L. rhamnosus CRL1505 (16, 20). [file Image_3.TIF]

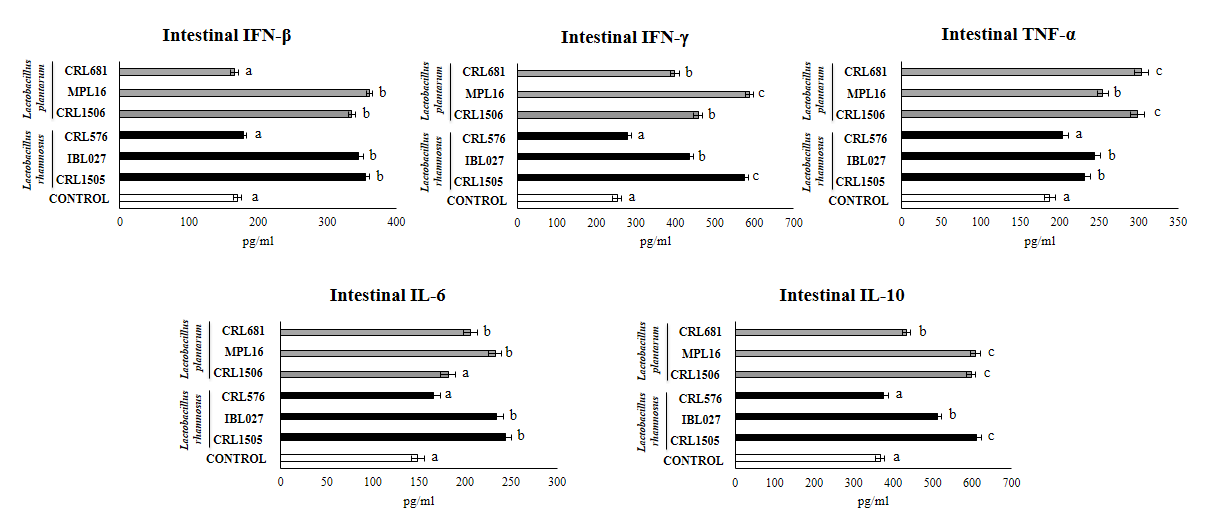

Supplement: FIGURE S4 — Levels of intestinal interferons (IFN-β and IFN-γ), proinflammatory cytokines (TNF-α and IL-6) and IL-10 in mice orally treated with Lactobacillus rhamnosus CRL1505, L. rhamnosus IBL027, L. rhamnosus CRL576, Lactobacillus plantarum CRL1506, L. plantarum MPL16, or L. plantarum CRL681 (108 cells/mouse/day for five consecutive days). Mice with no lactobacilli treatment were used as controls. Immune factors were evaluated on day 6. The results represent data from three independent experiments (n = 6 per group). Letters indicate significant differences (P < 0.05), a < b < c. [file Image_4.TIF]

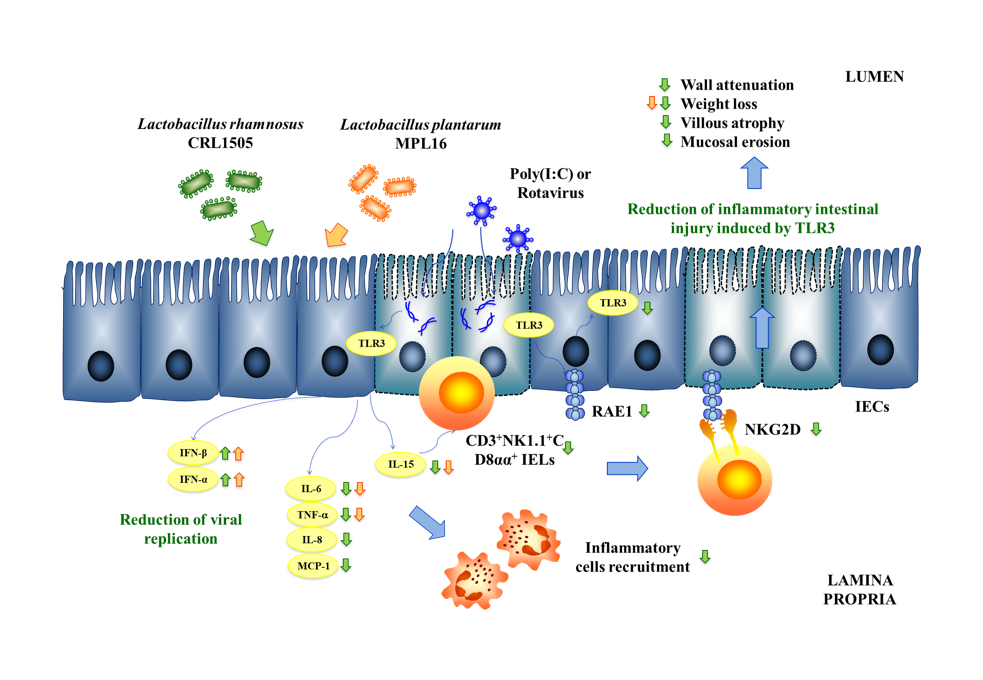

Supplement: FIGURE S5 — Global overview of the effect of L. rhamnosus CRL1505 and L. plantarum MPL16 on the intestinal inflammatory injury induced by poly(I:C) and mediated by the activation of CD3+NK1.1+CD8αα+ intraepithelial lymphocytes (IELs) that promote epithelial destruction through the RAE1–NKG2D interaction. Detailed immunomodulatory mechanisms were previously studied for L. rhamnosus CRL1505 (8). [file Image_5.TIF]

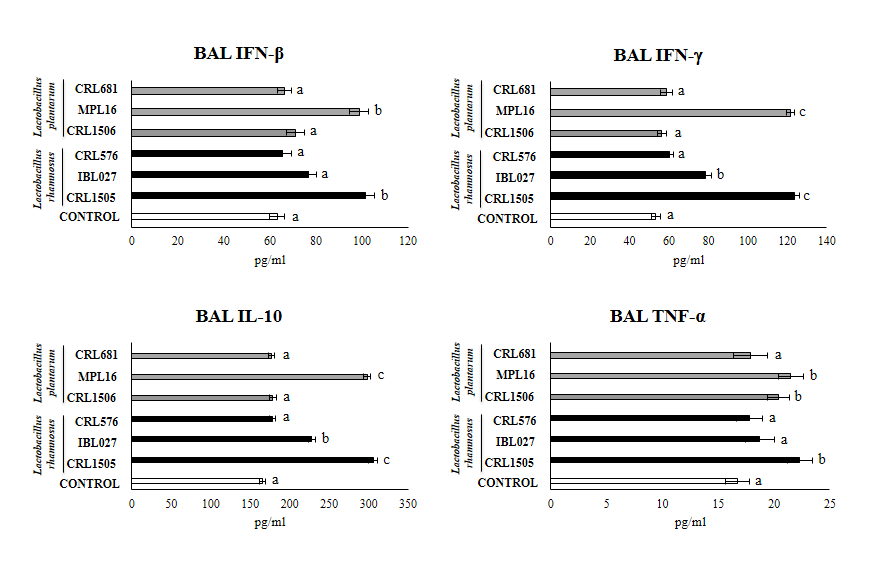

Supplement: FIGURE S6 — Levels of bronco-alveolar lavages (BAL) interferons (IFN-β and IFN-γ), TNF-α and IL-10 in mice orally treated with Lactobacillus rhamnosus CRL1505, L. rhamnosus IBL027, L. rhamnosus CRL576, Lactobacillus plantarum CRL1506, L. plantarum MPL16, or L. plantarum CRL681 (108 cells/mouse/day for five consecutive days). Mice with no lactobacilli treatment were used as controls. Immune factors were evaluated on day 6. The results represent data from three independent experiments (n = 6 per group). Letters indicate significant differences (P < 0.05), a < b < c. [file Image_6.TIF]

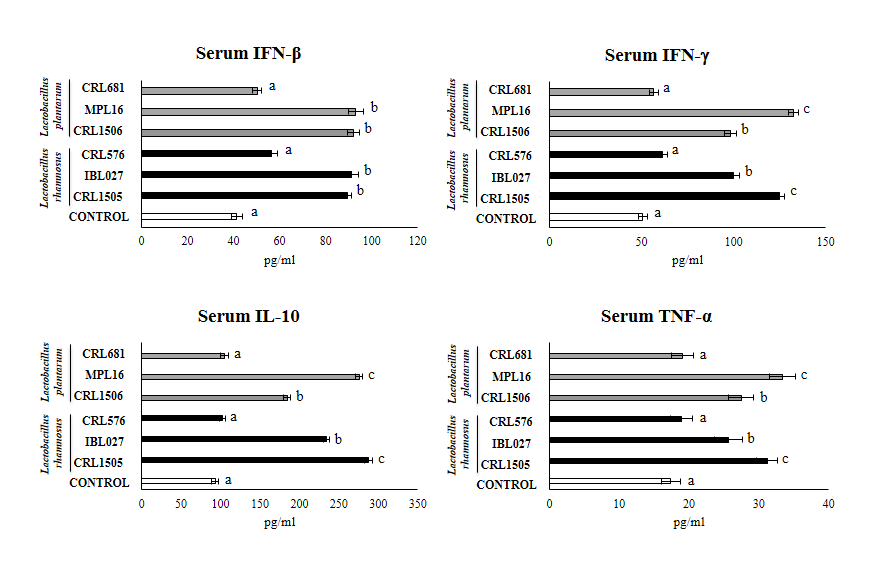

Supplement: FIGURE S7 — Levels of serum interferons (IFN-β and IFN-γ), TNF-α and IL-10 in mice orally treated with Lactobacillus rhamnosus CRL1505, L. rhamnosus IBL027, L. rhamnosus CRL576, Lactobacillus plantarum CRL1506, L. plantarum MPL16, or L. plantarum CRL681 (108 cells/mouse/day for five consecutive days). Mice with no lactobacilli treatment were used as controls. Immune factors were evaluated on day 6. The results represent data from three independent experiments (n = 6 per group). Letters indicate significant differences (P < 0.05), a < b < c. [file Image_7.TIF]

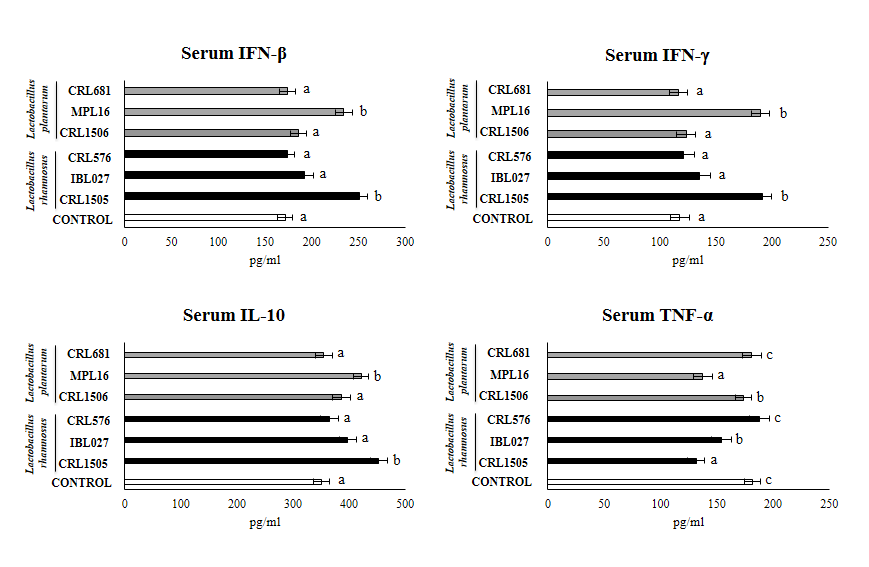

Supplement: FIGURE S8 — Levels of serum interferons (IFN-β and IFN-γ), TNF-α and IL-10 in mice orally treated with Lactobacillus rhamnosus CRL1505, L. rhamnosus IBL027, L. rhamnosus CRL576, Lactobacillus plantarum CRL1506, L. plantarum MPL16, or L. plantarum CRL681 (108 cells/mouse/day for five consecutive days), and then challenged by the nasal route with 250 μg of the viral molecular associated pattern poly(I:C) for three consecutive days. Mice with no lactobacilli treatment and challenged with poly(I:C) were used as controls. Serum immune factors were evaluated on day 2 after the last poly(:C) administration. The results represent data from three independent experiments (n = 6 per group). Letters indicate significant differences (P < 0.05), a < b < c. [file Image_8.TIF]

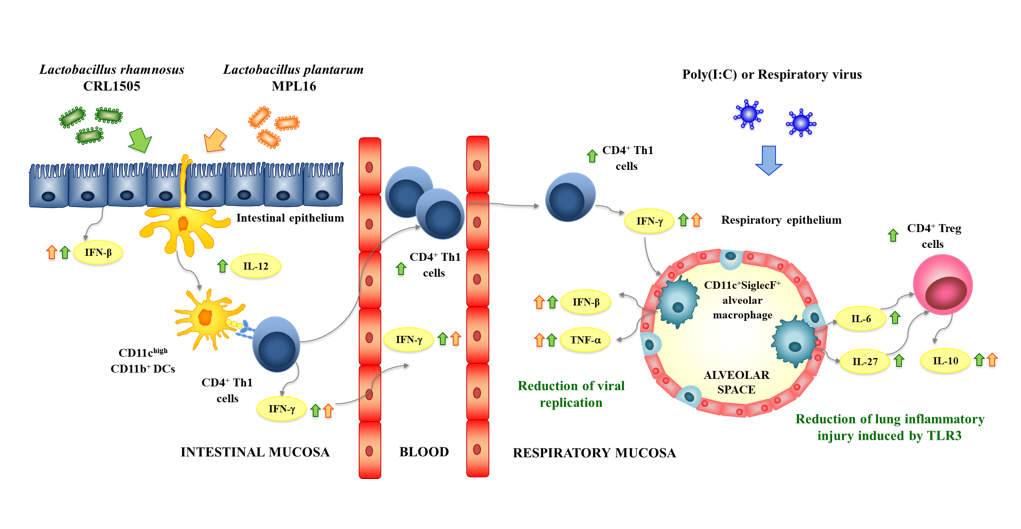

Supplement: FIGURE S9 — Global overview of the effect of L. rhamnosus CRL1505 and L. plantarum MPL16 on the respiratory innate immune response triggered by poly(I:C). Detailed immunomodulatory mechanisms were previously studied for L. rhamnosus CRL1505 (7, 9). [file Image_9.TIF]
